# Protective Immunity Induced by Immunization with Baculovirus, Virus-like Particle, and Vaccinia Virus Expressing the AMA1 of *Plasmodium berghei*

**DOI:** 10.3390/biomedicines10092289

**Published:** 2022-09-15

**Authors:** Min-Ju Kim, Ki-Back Chu, Hae-Ji Kang, Keon-Woong Yoon, Gi-Deok Eom, Jie Mao, Su-Hwa Lee, Jeeva Subbiah, Sang-Moo Kang, Eun-Kyung Moon, Fu-Shi Quan

**Affiliations:** 1 Department of Biomedical Science, Graduate School, Kyung Hee University, Seoul 02447, Korea; 2 Department of Medical Zoology, School of Medicine, Kyung Hee University, Seoul 02447, Korea; 3 Medical Research Center for Bioreaction to Reactive Oxygen Species and Biomedical Science Institute, School of Medicine, Graduate School, Kyung Hee University, Seoul 02447, Korea; 4 Center for Inflammation, Immunity, and Infection, Institute for Biomedical Sciences, Georgia State University, Atlanta, GA 30303, USA

**Keywords:** *Plasmodium berghei*, apical membrane antigen 1, virus-like particle, baculovirus, vaccinia virus, vaccine

## Abstract

Heterologous prime–boost immunization regimens using various vaccine platforms demonstrated promising results against infectious diseases. Here, mice were sequentially immunized with the recombinant baculovirus (rBV), virus-like particle (VLP), and recombinant vaccinia virus (rVV) vaccines expressing the *Plasmodium berghei* apical membrane antigen 1 (AMA1) for protective efficacy evaluation. The rBV_V_rVV heterologous immunization regimen elicited high levels of parasite-specific IgG, IgG2a, and IgG2b antibody responses in sera. Upon *P. berghei* challenge infection, proliferations of germinal center B cells in the inguinal lymph nodes, as well as blood CD4^+^ and CD8^+^ T cells were induced. More importantly, rBV_V_rVV immunization significantly diminished the parasitemia and prevented drastic bodyweight loss in mice post-challenge infection with *P. berghei*. Our findings revealed that immunization with rBV, VLP, and rVV expressing the AMA1 conferred protection against *P. berghei* infection, providing evidence for the potential implementation of this strategy.

## 1. Introduction

Malaria and malaria-related deaths remain a public health hazard. In the year 2018, an estimated total of 228 million cases of malaria were reported from around the world, with a large portion of this global malaria burden being attributable to India and sub-Saharan nations of Africa [1]. However, an efficacious vaccine for this deadly disease is commercially unavailable. RTS,S/AS01 (Mosquirix^TM^) is currently the most advanced malaria vaccine, but its suboptimal endpoint efficacy demonstrated in infants, which further waned over time, remains an issue that must be resolved [2,3]. Given the current situation, developing an efficacious malaria vaccine is of utmost priority as this will help reduce the socioeconomic burden around the globe and extend human longevity.

Apical membrane antigen 1 (AMA1) of *Plasmodium* spp. is an important merozoite-stage surface antigen that contributes to the parasitic invasion of red blood cells. To date, several vaccines based on the apical membrane antigen 1 (AMA1) have been tested at both pre-clinical and clinical levels. In clinical trials, the efficacy of AMA1-based protein subunit vaccines has been disappointing, and several factors such as antigenic polymorphism or deficient cellular immunity have been suggested [4]. For instance, immunization with the recombinant AMA1 protein fused to the merozoite surface protein 1 (MSP1) resulted in high antibody titers, but the generated antibodies failed to demonstrate biological activity, as indicated by the lack of parasite inhibition in vitro [5]. One potential solution to address the disappointing results from adjuvanted recombinant protein vaccines is the use of heterologous immunization. Viral vectors such as the adenovirus (Ad) and the recombinant vaccinia virus (rVV) modified vaccinia Ankara (MVA) strain are of particular interest. The protective efficacy of these vectored vaccines expressing a vast array of malaria antigens has been clinically assessed as early as 2005, with promising results [4]. For instance, the AMA1 gene of *P. falciparum* delivered using the Ad-MVA heterologous immunization strategy elicited functional T cell and antibody responses in mice and rabbits [6]. This vaccine demonstrated similar findings when tested in Phase Ia clinical trials, and parasite-inhibiting activity was confirmed [7]. Such an approach was also effective against *P. chabaudi* when tested in BALB/c mice [8].

While the heterologous immunization approach enhanced the vaccine efficacies of AMA1-based vaccines, the findings were not always consistent. *P. vivax* AMA1 immunization via recombinant protein and the Ad-vectored prime–boost regimen ensured potent antigen-specific antibodies and memory T cell responses [9]. However, vaccine-induced protection data upon challenge infection were not provided in this study, which precludes the drawing of conclusions regarding vaccine efficacy. In another study, heterologous immunization with vaccines expressing AMA1 and other erythrocytic or pre-erythrocytic antigens of *P. knowlesi* failed to elicit long-lasting protection in rhesus macaques [10]. Therefore, testing different vaccine platforms to boost the efficacy of heterologous immunization strategies is a necessity. Among the clinically tested vaccine platforms is the recombinant baculovirus (rBV). The prime and boost immunization of human subjects with rVV and the baculoviruses-derived gp160 protein of the human immunodeficiency virus induced high titers of virus-specific antibody responses [11,12,13]. Virus-like particles are also of keen interest for their high immunogenic properties. Nevertheless, studies investigating the efficacy of *Plasmodium* spp. AMA1-expressing rBVs or VLPs are extremely limited, and not a single study has attempted to investigate the enhanced efficacy induced through heterologous immunization involving rBV, VLP, and rVV.

In our previous study, mice were immunized with virus-like particles expressing the codon-optimized *P. berghei* AMA1 antigen, but VLP vaccine-induced protection was limited [14]. Interestingly, *P. berghei* possesses features that are similar to those of *P. falciparum,* which include parasite sequestration and possibly even the immune evasion mechanism [15,16]. These properties make *P. berghei* a valid candidate for use as an infection model organism. To improve the protective efficacy of the AMA1 VLPs, we employed a heterologous immunization approach by combining the VLP vaccine platform with the AMA1-expressing rBV and rVV viral vectors. We found that the rBV-VLP-rVV immunization strategy induced both humoral and cellular immunity, significantly reduced the parasite burden, and prevented drastic bodyweight loss in mice as compared to unimmunized mice.

## 2. Materials and Methods

### 2.1. Ethics Statement

All animal experiments in this study were carried out under the guidelines set out by Kyung Hee University IACUC, and the experimental protocols involving animals were approved (permit number: KHUIBC(SE)-19-034). Immunization and blood collection were performed under mild anesthesia, which was induced and maintained with ketamine hydrochloride and xylazine. All efforts were made to minimize the number of animals used in the experiment as well as their suffering.

### 2.2. Animals, Parasites, Cells, and Antibodies

Seven-week-old female BALB/c mice (*n* = 8 per group) were purchased from NARA Biotech (Seoul, Korea). BALB/c mice were infected to maintain the *Plasmodium berghei* ANKA strain, which was used for challenge infection. To generate recombinant baculovirus (rBV) and virus-like particles (VLPs), *Spodoptera frugiperda* insect cells (Sf9) were used and maintained using a serum-free SF900-II medium (Invitrogen, Carlsbad, CA, USA). CV-1 (ATCC, CCL-70) and Vero (ATCC, CCL-81) cell lines were used for the production of recombinant vaccinia virus (rVV) and were maintained using Dulbecco’s Modified Eagle Media (DMEM). Sera of *P. berghei*-infected mice were collected and used as polyclonal antibodies to detect *P. berghei* AMA1-specific responses. Horseradish peroxidase (HRP)-conjugated goat anti-mouse IgG, IgG1, IgG2a, and IgG2b were purchased from Southern Biotech (Birmingham, AL, USA).

### 2.3. Plasmodium Berghei Antigen Preparation

*P. berghei* ANKA strain antigen was collected and used as a coating antigen for the enzyme-linked immunosorbent assay (ELISA), as described previously [17]. Briefly, blood samples were collected from *P. berghei*-infected mice with parasitemia exceeding 20%. After low-speed centrifugation, sedimented red blood cells (RBCs) were lysed with 0.15% saponin diluted in equal volumes of phosphate-buffered saline (PBS) at 37 °C for 10 min. Parasites released from the RBCs were pelleted, washed with PBS, and then sonicated. After centrifugation, the supernatant fraction containing the crude lysate antigen was stored at −20 °C until use.

### 2.4. Generation of VLPs

*P. berghei* AMA1 VLPs were prepared, as described previously [14]. Briefly, the codon-optimized *P. berghei* AMA1 gene and influenza M1 were cloned into the pFastBac vector, which was subsequently transformed into DH10Bac competent cells. Bacmid DNA was collected and used to transfect Sf9 cells for rBV production. The Sf9 cells were co-infected with the AMA1 and M1-expressing rBVs to generate VLPs.

### 2.5. Generation of Recombinant Vaccinia Virus

To generate the *P. berghei* recombinant vaccinia virus, the codon-optimized *P. berghei* AMA1 gene purchased from GenScript (Piscataway, NJ, USA) was cloned into the pRB21 vaccinia virus transfer vector. CV-1 cells were cultured in DMEM (WELGENE, Daegu, Korea) supplemented with 10% heat-inactivated FBS and 1% penicillin/streptomycin. A confluent monolayer of CV-1 cells seeded in 12-well plates was infected with the vRB12 vaccinia virus strain in serum-free DMEM media at 37 °C for 1 h. Recombinant pRB21 plasmid expressing the AMA1 gene was transfected into CV-1 cells using Lipofectamine LTX/PLUS™ (Life Technologies, Inc., Carlsbad, CA, USA), and cells were incubated at 37 °C for 3 days. Recombinant vaccinia virus was harvested after 3 days and centrifuged at 2000× *g* for 10 min. After discarding the supernatant and resuspending the pelleted cells in DMEM media, the cells were subjected to 3 freeze–thaw cycles using liquid nitrogen and a 37 °C water bath. The cells were sonicated for rVV acquisition, and the rVVs were further amplified by infecting monolayers of CV-1 cells at 37 °C for 3–4 days.

### 2.6. Recombinant Vaccinia Virus Plaque Assay

Recombinant vaccinia virus titers were determined using Vero cells. Briefly, Vero cells were seeded in 12-well cell culture plates. At 90–100% confluency, the cells were infected with serially diluted rVVs in serum-free DMEM media at 37 °C for 1 h. The overlay media was made with DMEM media containing 2% FBS and 1% noble agarose (BD Bioscience, Franklin Lakes, NJ, USA). After incubating for 1 h, serially diluted rVVs were aspirated, and overlay media was added to each well before incubating at 37 °C. Infected cells were maintained until viral plaques became visible. To enhance plaque visibility, neutral red dye (Sigma Aldrich, St. Louis, MO, USA) was diluted 100-fold in DMEM media containing 2% agarose and then added to each well prior to incubation at 37 °C; the enlarged plaques were counted to determine the rVV titers.

### 2.7. Recombinant Baculovirus Plaque Assay

Viral titers of recombinant baculoviruses were determined, as previously described [18]. Briefly, Sf9 cells seeded in 96-well plates were infected with serially diluted rBVs and incubated for 1 h. After incubation, the rBVs were removed and overlaid with 0.8% noble agar and incubated at 27 °C for 3 days. Upon agar removal, the cells were fixed and blocked with 3% skim milk before incubation with polyclonal *P. berghei* antibody. HRP-conjugated mouse IgG was used as a secondary antibody, and 3,3-diaminobenzidine (DAB) chromogenic substrate was used to visualize plaques, which were counted under the microscope.

### 2.8. Immunization and Challenge Infection

The mice were intramuscularly immunized with *P. berghei* AMA1 rBV (1.2 × 10^5^ pfu/mouse), AMA1 VLPs (100 ug/mouse), or AMA1 rVV (1.3 × 10^8^ pfu/mouse) at weeks 0, 4, and 8. Heterologous immunization (rBV_V_rVV) refers to the priming of mice with the AMA1 rBVs and subsequently immunizing them with the AMA1 VLPs and rVVs. Four weeks after the final immunization, the mice were infected with 5.0 × 10^3^ *P*. *berghei* parasites immersed in 100 ul PBS through the intraperitoneal route. At 6 days post-infection (dpi), 4 mice from each group were sacrificed for blood, spleen, and inguinal node (ILN) sampling. The remaining 4 mice were used to monitor changes in bodyweight and survival. Mice that lost 20% of their initial body weight were humanely euthanized.

### 2.9. Antibody Responses in Sera

Sera were collected 4 weeks after each immunization using retro-orbital plexus puncture. Naïve mice sera were used as a negative control. Collected sera were used to determine antibody responses against *P. berghei* antigen, AMA1 VLP, and M1 VLP using the enzyme-linked immunosorbent assay (ELISA). Briefly, 96-well immunoplates were coated with *P. berghei* antigens (2 μg/mL), AMA1 VLPs (0.5 μg/mL), or M1 VLPs (0.5 μg/mL) in carbonate coating buffer and blocked with 5% gelatin in 0.05% Tween-20 in 0.1 M PBS. Sera from mice were serially diluted in PBS, added to respective wells, and then incubated at 37 °C for 1.5 h. HRP-conjugated goat anti-mouse IgG, IgG1a, IgG2a, and IgG2b (100 μL/well, diluted 1:2000 in PBS) were used to determine the levels of antibody responses.

### 2.10. Flow Cytometry

Flow cytometry was performed to assess the T and B cell populations in the blood and inguinal lymph nodes (ILN) of immunized mice. Blood samples and ILN cells were harvested from mice 4 weeks after the final immunization and at 6 dpi. The cells were stimulated with sonicated *P. berghei* whole antigen at a concentration of 0.5 μg/mL at 37 °C for 2 h. Afterward, the cells were resuspended in FACS staining buffer (2% bovine serum albumin and 0.1% sodium azide in 0.1 M PBS) at 4 °C for 15 min with Fc Block (clone 2.4G2; BD Biosciences, San Jose, CA, USA). After Fc receptor blocking, the cells were stained with CD3e (PE-Cy5), CD4 (FITC), CD8a (PE), B220 (FITC), and GL7 (PE) fluorophore-conjugated antibodies. The CD4^+^ T cell, CD8^+^ T cell, and germinal center B cell populations were analyzed, as previously described [19].

### 2.11. Parasitemia

Blood samples were collected at regular intervals after challenge infection to determine parasitemia. Briefly, 2 μL blood samples were stained by mixing with PBS solution containing heparin and 1 μL SYBR Green I (Invitrogen, Carlsbad, CA, USA). After incubating at 37 °C for 30 min, the stained blood samples were analyzed by flow cytometry as previously described [14,20].

### 2.12. Statistics

All parameters were recorded for individuals within all groups. All data were presented as mean ± SD, and statistical significances between groups were analyzed by one-way analysis of variance (ANOVA) and Student’s *t*-test using GraphPad Prism version 6.0 (San Diego, CA, USA). The statistical significances between the means were denoted using asterisks (* *p* < 0.05, ** *p* < 0.01, *** *p* < 0.001).

## 3. Results

### 3.1. Characterization of Vaccines

**Figure 1:**
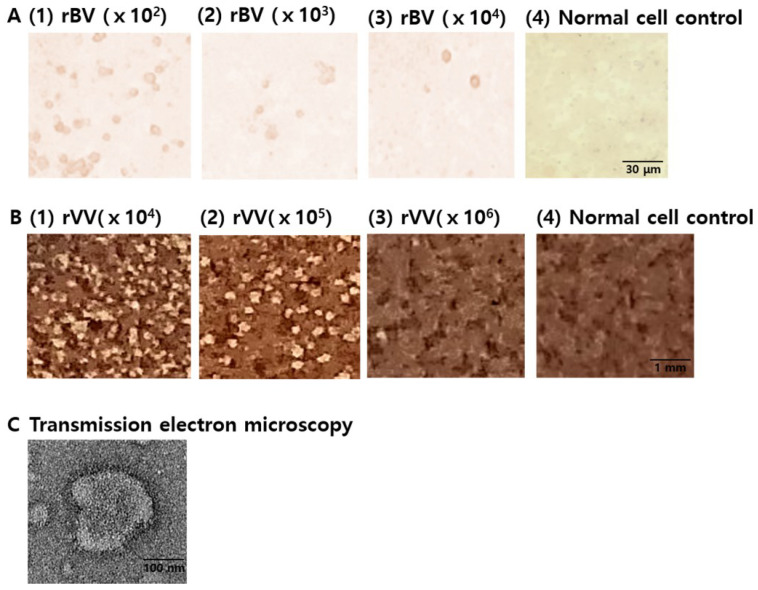
Vaccine Characterization. *P. berghei* AMA1 rBV, rVV, and VLPs were characterized through plaque assay and TEM. Sf9 cells were infected with serially diluted AMA1 rBVs, and plaques were visualized as brown precipitates (**A**). White AMA1 rVV plaques in Vero cells were observed (**B**). Morphology of the generated AMA1 VLPs was visualized under the transmission electron microscope (TEM) (**C**).

Codon-optimized *P. berghei* AMA1 rBV, rVV, and VLPs were generated and characterized. Serially diluted rBVs were used to transfect Sf9 cells, and immunostaining via DAB substrate was used to identify infected cells. At 100-fold dilution, noticeable amounts of brown precipitates were observed, which waned in number with further dilutions. On the contrary, the brown precipitates were not observed in the normal cell control group (Figure 1A). Similar to the rBVs, *P. berghei* AMA1 rVVs were characterized by plaque assay using Vero cells. As expected, white plaques denoting cellular lysis incurred by the rVVs were visible even at 10^4^ dilution, with their numbers gradually diminishing with further dilutions, whereas plaques were undetected in normal cell control (Figure 1B). The successful generation of AMA1 VLPs was confirmed by transmission electron microscopy (TEM, Figure 1C).

### 3.2. IgG Antibody Response against VLP Antigens in Sera

**Figure 2:**
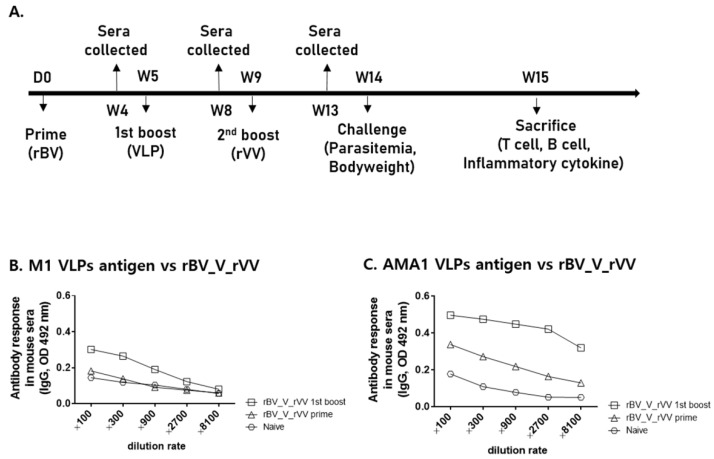
IgG antibody response against AMA1 VLP or M1 VLP antigens in sera. Immunizations and challenge infections of mice were performed as scheduled (**A**). Sera were collected 4 weeks after each immunization and were used to determine the antibody response against M1 and AMA1 VLPs. The M1 VLP-specific antibody response (**B**) and the vaccine-induced antibody response to AMA1 VLPs (**C**) were measured to confirm the successful immunization of mice. Data are presented as mean ± SD.

Mouse immunization with the *P. berghei* AMA1 vaccines and challenge infection were performed as scheduled (Figure 2A). To confirm the successful immunization of mice, antibody responses against the AMA1 VLPs and M1 VLPs were assessed. Sera were acquired 4 weeks after prime and 1st boost immunizations. AMA1 rBV priming did not elicit M1-specific antibody response, whereas a noticeable increase was observed upon boost immunization with AMA1 VLP (Figure 2B). However, rBV priming did elicit antigen-specific responses against AMA1 VLPs. Boost immunization with the AMA1 VLPs further enhanced the antigen-specific antibody responses from sera (Figure 2C). Elevated AMA1-specific antibody responses were maintained at high levels but rapidly waned after a 900-fold serial dilution.

### 3.3. Immunizations with rBV_V_rVV Elicited Parasite-Specific IgG and IgG Subclass Responses in Sera

**Figure 3:**
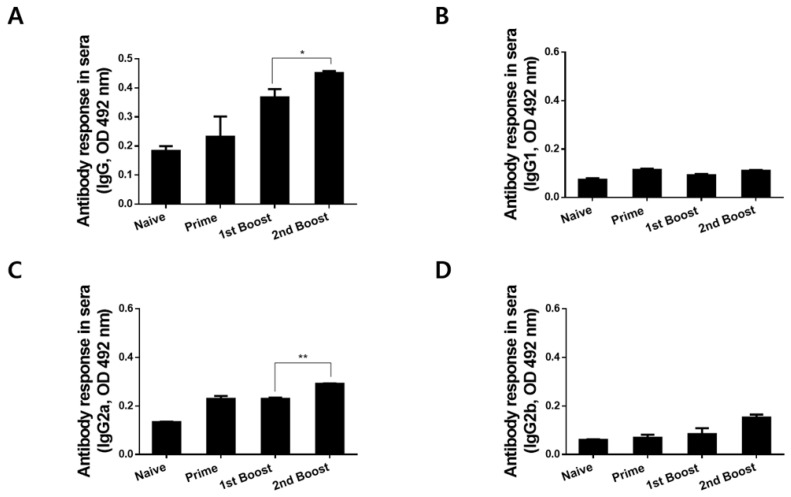
*P. berghei*-specific IgG, IgG1, IgG2a, and IgG2b responses in sera. Mice were subjected to heterologous immunization with the AMA1 vaccines. Sera were collected 4 weeks after each immunization and used to assess *P. berghei*-specific IgG (**A**), IgG1 (**B**), IgG2a (**C**), and IgG2b (**D**) antibody responses via ELISA. Data are presented as mean ± SD (* *p* < 0.05, ** *p* < 0.01).

To determine *P. berghei*-specific antibody responses, the sera from immunized mice were collected 4 weeks after each immunization. Subsequent immunizations contributed to enhancing the parasite-specific antibody inductions, as evidenced by the highest antibody levels being detected after the 2nd boost immunization. While parasite-specific antibody inductions were negligible upon rBV prime, subsequent boost immunizations induced significantly high levels of *P. berghei*-specific IgG (Figure 3A, * *p* < 0.05) and IgG2a (Figure 3C, ** *p* < 0.01). However, the IgG1 (Figure 3B) and IgG2b (Figure 3D) inductions post-immunization were negligible.

### 3.4. Potent Germinal Center B Cell Responses Are Induced in Inguinal Lymph Node

**Figure 4:**
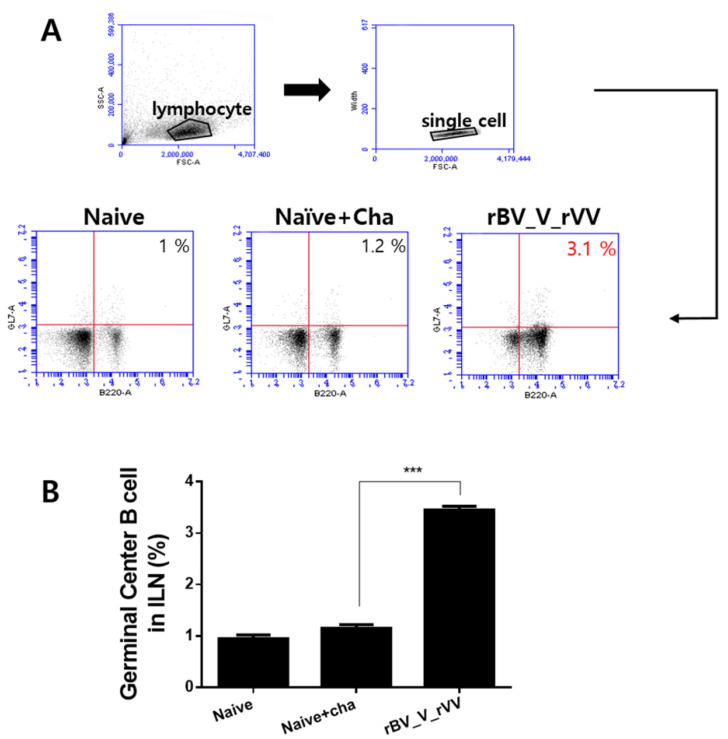
Germinal center B cell response in inguinal lymph node. GC B cell responses in the ILN were assessed using flow cytometry at 6 dpi. Acquired single-cell populations were gated, and representative GC B cell scatter plots for each group are shown (**A**). GC B levels of each group in the ILN (**B**) are presented as mean ± SD (*** *p* < 0.001).

The inguinal lymph node (ILN) cells of mice were collected at 6 dpi with *P. berghei* to detect the levels of germinal center (GC) B cells. The single-cell population of lymphocytes was gated and analyzed via flow cytometry (Figure 4A). Compared to the naïve or naïve+cha control groups, the heterologous immunization regimen enhanced the GC B cell propagation in the ILN. An approximately 3-fold increase in the GC B cells was observed in immunized mice regardless of the immunization regimen (Figure 4B, *** *p* < 0.001).

### 3.5. Immunizations with rBV_V_rVV Induced Potent CD8^+^, but Not CD4^+^ T Cell Responses in the Blood

**Figure 5:**
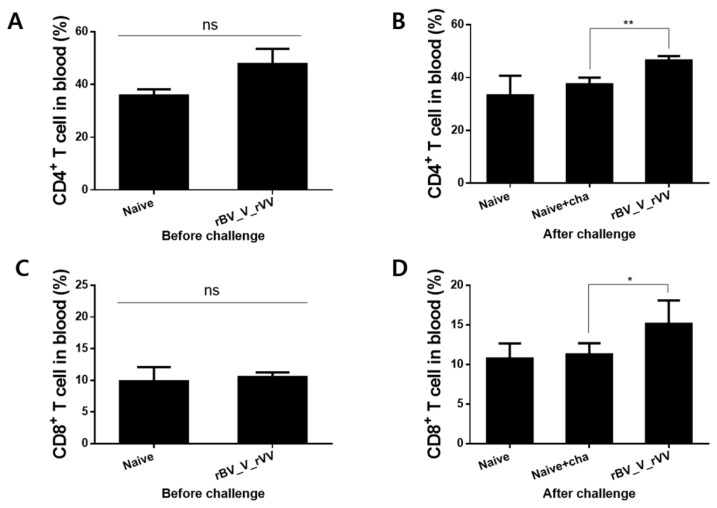
T cell response in the blood. Blood samples were collected at designated time points and analyzed by flow cytometry to assess CD4^+^ and CD8^+^ T cells. CD4^+^ T cells from the blood of mice collected at 4 weeks after the final immunization (**A**) and at 6 dpi (**B**) were quantified. CD8^+^ T cell propagations were also evaluated from the blood of mice at 4 weeks post-second boost immunization (**C**) and also at 6 dpi (**D**). Data are presented as mean ± SD (ns: no significance, * *p* < 0.05, ** *p* < 0.01).

To compare the vaccine-induced changes to the CD4^+^ and CD8^+^ T cell populations, peripheral blood samples were collected from mice at 2 different time points: at 4 weeks after the 2nd boost immunization and at 6 dpi. Differences in the CD4^+^ T cell populations were negligible prior to challenge infection (Figure 5A). Upon challenge infection, there was a marked increase in CD4^+^ T cell population from the vaccinated mice (Figure 5B, ** *p* < 0.01). A similar trend was also observed in the CD8^+^ T cell population in the blood. While significant differences in CD8^+^ T cell populations were not detected across all groups before challenge infection (Figure 5C), challenging the mice with *P. berghei* resulted in increased CD8^+^ T cell populations in the blood of vaccinated mice. Similar to the findings observed from CD4^+^ T cells, heterologous immunization-induced changes to the CD8^+^ T cells were statistically significant (Figure 5D, * *p* < 0.05).

### 3.6. Immunizations with rBV_V_rVV Lessened Parasitemia and Bodyweight Reduction in Immunized Mice

**Figure 6:**
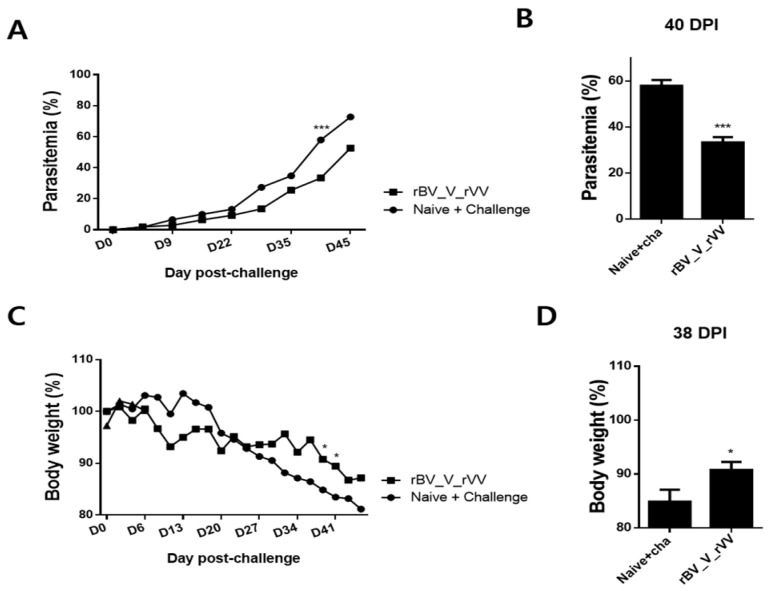
Parasitemia and body weight changes. Mice were monitored for 45 days post-infection. Parasitemia and changes in bodyweight were assessed every 3–5 days. Parasitemia was evaluated in the blood samples from mice at regular intervals (**A**). Blood parasitemia in mice subjected to *heterologous* immunizations was compared to the control group at 40 dpi (**B**). Bodyweight reductions were recorded regularly for 45 days post-challenge infection (**C**). Bodyweight data recorded at 38 dpi were used to compare differences across the groups (**D**). Data are presented as mean ± SD (* *p* < 0.05, *** *p* < 0.001).

After parasite challenge infection, the mice were monitored for 45 days. Parasitemia in the blood and bodyweight changes were measured every 3–5 days to determine vaccine efficacies. Noticeable increases in parasitemia began to appear at 30 dpi. While progressively increased parasitemia was observed for all groups, this occurred to a lesser extent in the vaccinated mice (Figure 6A, *** *p* < 0.001). At 40 dpi, heterologous immunization resulted in a nearly twofold reduction in parasitemia compared to the unimmunized control group (Figure 6B, *** *p* < 0.001). From 6 dpi onwards, gradual bodyweight loss became evident in challenge-infected mice. Bodyweight fluctuations were observed from heterologously immunized mice, but the trend seems to be gradual bodyweight reduction over the span of 45 days (Figure 6C, * *p* < 0.05). In particular, the bodyweights were significantly different at 38 dpi in immunized mice as compared to the unimmunized control (Figure 6D, * *p* < 0.05).

## 4. Discussion

A wide array of vaccine studies have reported the benefits of using a heterologous prime–boost regimen, which provided enhanced protection against infectious diseases when compared to a homologous counterpart. Here, we demonstrated that the heterologous immunization strategy utilizing rBV, VLP, and rVV can confer protection against *P. berghei* infection in mice.

Several conflicting reports regarding heterologous immunization against malaria have been reported. For instance, in the case of chimeric VLPs constructed using the T cell or B cell epitopes of *P. yoelii* CSP, the DNA-VLP prime–boost strategy was a more efficient inducer of cellular immunity than homologous VLP immunization [21]. However, because our study did not involve a DNA vaccine component, directly comparing our results with the aforementioned study is difficult. Heterologous immunization with the adenovirus and MVA vector vaccines expressing the multiple epitope thrombospondin adhesion protein was reported to be safe and immunogenic in West African children [22]. Contrastingly, different results were documented in a heterologous immunization study comparing the protective efficacy induced by multiple vaccine platform combinations. Priming BALB/c mice with the Ad vector expressing the *P. vivax* cell-traversal protein for ookinetes and sporozoites (PvCelTOS), then boosting with a different vaccine platform such as MVA or VLP that expressed an identical antigen failed to induce significant protection against transgenic *P. berghei* sporozoite challenge infection [23]. While it may appear as if heterologous immunization involving VLPs is ineffective, one must take into account the differences in the cell line sources used in the construction of VLPs. Here, we used insect cells to generate our VLPs, whereas the study above used bacteria. Post-translation modifications (PTMs) that occur in these two expression systems are strikingly different, and since these PTMs can hamper the antigen’s immunogenicity, it is highly plausible that this aspect contributed to the study’s outcome. Evidently, when mice were homologously immunized with yeast-derived VLPs, which has substantially different PTM than bacteria, the efficacies were much greater than those elicited by Ad-MVA heterologous immunization [24].

Multitudes of studies have investigated the efficacy of VLPs or MVA strain vaccinia virus-based vaccines against malaria using animal models. However, much remains to be discovered for rBV-based malaria vaccines. Previously, baculoviruses were simply utilized as a tool for protein expression, and the resulting proteins were subsequently used for immunization in animal models. For instance, in one earlier study, the authors used both baculovirus-expressed protein and recombinant vaccinia viruses along with a panel of H-2 congenic mice to demonstrate that the circumsporozoite protein of *Plasmodium* spp. was weakly immunogenic [25]. AMA1 is no exception as immunizing mice with baculovirus-expressed AMA1 contributed to protection against *P. chabaudi* infection [26]. Though limited in number, a few studies did utilize rBVs as a vaccine platform, as we have done in the present study. The intramuscular immunization of mice with baculovirus vaccines expressing the circumsporozoite protein (CSP) of *P. falciparum* while also displaying the identical antigen on the virion surface elicited a strong immune response [27]. Immunizing mice with rBVs expressing the CSP of *P. berghei* conferred 60% protection in mice upon sporozoite challenge infection [28]. Similarly, immunization with the baculoviruses expressing the *P. vivax* P25 and CSP conferred 43% protection against the transgenic *P. berghei* sporozoites challenge infection in mice, while P25 or CSP-expressing rBVs demonstrated 3% and 12% protection, respectively [29]. The protective efficacy of rBVs expressing the CSP antigen as a transmission-blocking vaccine was further evaluated by the same research group using various methods. These strategies included incorporating the gene encoding the decay-accelerating factor that confers complement resistance in vivo [30], co-immunization with IL-12-expressing baculovirus vectors [31], and using an adenovirus-baculovirus heterologous prime–boost regimen [32]. As evidenced by these previous research papers, rBV-based vaccines can be efficient inducers of protective immunity against malaria, and their development as a vaccine candidate is feasible.

Priming mice with the AMA1-rBVs failed to induce a significant increase in parasite-specific antibody responses in the sera. Though speculative, there are two possible explanations for this phenomenon. First, it is plausible that the antibodies raised via rBV immunization may have elicited neutralizing antibody titers that impeded rBV-mediated gene expression and transduction in mice. Though the exact mechanism of this repression has not been fully elucidated, it is thought to revolve around an antagonistic relationship involving the baculoviral gp64 protein [33]. Second, the immunization dose of rBVs used in the present study may have been suboptimal. In other rBV vaccine studies, immunization doses that elicited protection against lethal challenge infection with viral pathogens generally exceeded 10^7^ pfu [34,35]. As the immunization dose used to prime mice in the present study was 2 orders of magnitude lower than the dose used in other rBV vaccine studies, this consequently led to weakened antibody induction.

Numerous studies have suggested that the IgG2a and IgG2b subclasses engage in complement-fixing activity to elicit protection upon infection with *Plasmodium* spp. [36]. However, given that murine complement fixation levels are significantly lower than those of humans and other mammals [37], accurately assessing the protection contributed by the enhanced levels of these IgG subclasses observed in our study is difficult. Previously, VLPs were shown to be equally effective inducers of cytotoxic T lymphocyte responses regardless of homologous or heterologous immunization methods [38], and this contributed to enhancing the CD8^+^ T cell circulation in the blood of mice. Although memory T cell responses were not assessed in the present study, our previous findings involving MSP-8 and MSP-9 VLP vaccines reported a potent induction of memory T cell responses following immunization [39,40]. This is also true for various vectored-vaccine platforms that utilized an immunization strategy similar to that incorporated in the present study [41]. Based on these previous reports, it is highly plausible that the vaccines used in this study may have also generated memory T cells in immunized mice to an extent.

While significant reductions in parasitemia and in bodyweight loss were observed in immunized mice, improving the protective efficacy of our vaccines through further studies is a necessity. One such method is by using a different strain of the vaccinia virus. Interestingly, several studies have reported that the strain of vaccinia virus used as a vaccine has a large impact on protection in vivo. For example, vaccines based on the Western Reserve strain of vaccinia virus conferred protection against anthrax in mice but not in guinea pigs, whereas protection was not elicited in any of the experimental animals immunized with the Connaught strain of vaccinia virus-based vaccines [42]. This phenomenon is thought to arise from the genetic differences in the vaccinia virus strains that create deviations in their ability to downmodulate cell-mediated immune responses in animals, which ultimately influences their virulence [43]. In conclusion, our findings demonstrated that heterologous immunization with rBV_V_rVV can be protective in mice.

## Data Availability

Data supporting the findings of this study are available within the article.
